# Xiaochaihutang Inhibits the Activation of Hepatic Stellate Cell Line T6 Through the Nrf2 Pathway

**DOI:** 10.3389/fphar.2018.01516

**Published:** 2019-01-07

**Authors:** Rui Hu, Wei-yi Jia, Shang-fu Xu, Zhi-wei Zhu, Zhi Xiao, Shou-yang Yu, Jin Li

**Affiliations:** ^1^Research Center for Medicine and Biology, Zunyi Medical University, Zunyi, China; ^2^Key Laboratory of Brain Science, Zunyi Medical University, Zunyi, China; ^3^Key Laboratory of Basic Pharmacology of Ministry of Education and Joint International Research Laboratory of Ethnomedicine of Ministry of Education, Zunyi Medical University, Zunyi, China

**Keywords:** Xiaochaihutang, hepatic stellate cells, Nrf2 pathway, hepatic fibrosis, serum pharmacology

## Abstract

Xiaochaihutang (XCHT) is one of classic prescriptions in Treatise on Febrile Diseases in China which was reported to have the effect of anti-hepatic fibrosis *in vivo*. Activation of hepatic stellate cells (HSCs) is now well established as a central driver of fibrosis in liver injury. Nuclear factor erythroid 2-related factor 2 (Nrf2) is an important element for anti-oxidative damage which is one of the key factors responsible for occurrence. This study was to investigate the effect of XCHT compound serum on HSCs activation and focus on the Nrf2 pathway. Rats in treatment groups were given the appropriate doses of XCHT granules (5 g/kg) and Silybin (50 mg/kg) for 6 days, and the serum were obtained. The compound serum was used to intervene HSCs. The results found that XCHT compound serum significantly inhibited the proliferation of HSCT6 cells. The number of α-SMA positive stained cells in HSCT6 cells and the content of Collagen type I (collagen-I) in supernatant were significantly decreased indicating suppression of activated HSCs. Compared with the control group, the nuclear transcription of Nrf2 and the expressions of Nqo1, GCLC, and GCLM were significantly increased in XCHT group. However, the effects of XCHT were inhibited in Nrf2-siRNA transfected HSCT6 cells. These studies demonstrated that XCHT could inhibit HSCT6 cell proliferation and activation. The mechanism might be related to up-regulation of the Nrf2 pathway against oxidative stress.

## Introduction

Liver fibrosis is the rate-limiting step in patients with liver disease on pathological transformation and mortality (Gandhi, 2017). The excessive activation and proliferation of hepatic stellate cells (HSCs) give rise to extracellular matrix proteins during the pathogenesis of hepatic fibrosis, initiating matrix deposition in the liver (Campana and Iredale, 2015). And repressing proliferation and activation of HSCs may be the potential strategy for hepatic fibrosis therapy.

Notably, more and more reports suggested that the pathogenesis of liver fibrosis was related to oxidative stress, which was marked by the intracellular accumulation of excessive reactive oxygen species (ROS), and the compelling evidence shows the involvement of ROS in the development of liver fibrosis inducing HSCs activation and fibrogenic potential (Novo and Parola, 2008; Novo et al., 2011; Puche et al., 2013). Nuclear factor erythroid 2-related factor 2 (Nrf2) is a key regulator in the redox balance (Cao et al., 2016). Under basic conditions, Nrf2 is constantly ubiquitinated through Kelch-like ECH-associated protein1 (Keap1) and degraded in the proteasome (Tong et al., 2006). Nrf2 separates from Keap1 when exposures to oxidative stress, binds to the antioxidant element (ARE) after translocating into the nucleus, and regulates antioxidant enzyme genes expression such as quinine oxidoreductase 1 (Nqo1), heme oxygenase 1 (HO-1), and GSH synthesis (GCLM, GCLC) mediated by ARE (Kaspar et al., 2009; Copple, 2012; Gum and Cho, 2013; Liu et al., 2013; Li et al., 2014d). It was found that Nrf2 regulated the activity of the antioxidant enzymes and the expression of down-stream genes to protect against CCl_4_-induced liver fibrosis (Li et al., 2017).

Xiaochaihutang (XCHT, Sho-Saiko-to, SST in Japan), a classic prescriptions of Traditional Chinese Medicine (TCM), is widely used for hepatic protection, analgesic treatment, anti-inflammation, and antipyretic. It was found that XCHT had a liver protective effect (Li, 2000; Jiang et al., 2007). Our previous study demonstrated that XCHT had a therapeutic effect on hepatic fibrosis induced by CCl_4_ in rats, and the mechanisms might be the protection against oxidative stress via regulation of Nrf2 pathway, making further efforts to inhibit the activated HSCs (Li et al., 2017). However, there is few study on the antifibrotic effect of XCHT directing on HSCs (Li et al., 2017; Hu et al., 2018).

Therefore, the present study aimed to investigate the pharmacological effects of XCHT-inhibited cell activation in a rat hepatic stellate cell line T6, HSCT6 cells, and subsequently delineate the underlying mechanisms in HSCs.

## Materials and Methods

### Drugs

Xiaochaihutang is a traditional Chinese Medicine. Xiaochaihu granule was purchased from Baiyunshan Guanghua Pharmaceutical Co., Ltd. (approval number K13711, Guangzhou, China), which has been embodied in the Chinese Pharmacopeia Committee of China (2015). The Xiaochaihu granule composition were *Bupleurum falcatum* L. (1.5 g), *Scutellaria baicalensis* Georgi (0.56 g), *Codonopsis pilosula* Franch (0.56 g), *Pinellia temata* Breitenbach (0.56 g), *Glycyrrhiza glabra* L. (0.56 g), *Zingiber officinale* Roscoe (0.56 g), and *Zizyphus vulgaris* Lam. (0.56 g) and excipients (5.14 g) (Pharmacopeia Committee of China, 2015). Baicalin is the index of content determination of Xiaochaihu granule. The content of baicalin was detected by high performance liquid chromatography (HPLC) and the results (24.307 mg/10 g) met the requirement of “No less than 20 mg/10 g” (Pharmacopeia Committee of China, 2015; Supplementary Figure S1).

### Preparation of XCHT Compound Serum

Animal experiment was performed in accordance with the Guide for the Care and Use of Laboratory Animals. Sprague-Dawley rats weighing 180–220 g (Experimental Animal Centre of the Third Military Medical University) were used. Rats were randomly divided into three groups: control group (*n* = 20), XCHT treatment groups (5 g/kg, *n* = 20), and Silybin treatment group as positive drug group (50 mg/kg, *n* = 20). Serum pharmacology as a new experiment method in *in vitro* pharmacology research, is gradually widely used in the field of traditional Chinese Medicine compound pharmacodynamics (Guo et al., 2017). According to the clinical application of dosage (Su et al., 2014) and the dose conversion from human to rat, the dosage of Xiaochaihu granule was 5 g/kg (equivalent to 1.5 g/kg of Xiaochaihutang). After treatment for 6 days, all rats were sacrificed and the blood were obtained. Standing at room temperature for 2 h, the blood was centrifuged at 860 × *g* for 15 min. The serum was separated and mixed with the serum of the same group. The serum was inactivated by a water bath at 56°C for 30 min, filtered through a 0.22 μm microporous membrane filter, and stored at -20°C refrigerator (Iwama et al., 1987).

### Cell Culture

Hepatic stellate cell line T6 (HSCT6) was derived from rat normal liver (KunMing Cell Bank, Chinese Academy of Sciences. KunMing Cell Bank number: KCB200703YJ). Cells were cultured in Dulbecco’s modified Eagle’s medium (DMEM/HIG GLUCOSE, HyClone, Utah, United States) supplemented with 10% (v/v) fetal bovine serum (FBS, Gibco, Australia), 100 μg/mL streptomycin (Sigma-Aldrich, United States), 100 U/mL penicillin (Sigma-Aldrich, United States), at 37°C in a humidity atmosphere of 5% (v/v) CO2.

There were four groups: XCHT compound serum group (XCHT group), Silybin compound serum group (Silybin group), Control-serum group (Control group), and the vehicle control group. The vehicle control group was cultured in DMEM supplemented with 10% (v/v) fetal bovine serum.

### Cell Viability Analysis

Hepatic stellate cell line T6 cells with a density of 5 × 10^3^ cells/well were evenly spread and incubated in a 96-well plate for 24 h and treated with different concentrations of XCHT compound serum [2.5, 5, and 10% (v/v)] for a further 24, 36, 48, and 60 h. The Cell counting Kit-8 (CCK8, Beyotime, China) was used to test the viability of HSCT6 cells. The CCK8 assay was used as originally described by the instruction. Briefly, the cell cultures were in-cubated with CCK8 (10 μL/well) for 1 h at 37°C, and absorbance of the lysates was measured spectro-photometrically at 450 nm. The results were referred to the absorbance of samples not treated by any agent, which was taken as 100% viability value. Under the incubation conditions used in the experiments as usual, none of the compounds added to the cell cultures affected the outcome of the CCK8 assay (data not shown).

### Enzyme-Linked Immunosorbent Assay

Hepatic stellate cell line T6 cells with a density of 2 × 10^5^ cells/well were evenly spread and incubated in a six-well plate for 24 h and treated with 10% (v/v) concentrations of XCHT compound serum for a further 60 h. The amount of collagen-I in the supernatant of HSCT6 cells were measured with commercial kits (Jianglaibio, Shanghai, China).

### Immunofluorescence Microscopy

Hepatic stellate cell line T6 cells were cultured on glass coverslips for 24 h and treated with XCHT compound serum for a further 24 h. Coverslips were wash with PBS, fixed in 4% (v/v) paraformaldehyde for 20 min at room temperature, washed, prior to detergent extraction with 0.3% (v/v) Triton X-100 for 10 min at 4°C. Coverslips were saturated with PBS containing 5% (v/v) Normal goat serum for 1 h at room temperature. Next, cells were incubated with the specific primary antibody for α-SMA for 1 h, washed, and incubated with secondary antibody. Finally, cells were stained for 30 min at room temperature with 4,6-diamidino-2-phenylindole (DAPI). Slides were viewed with OLYMPUS IX73 microscope.

### RT-PCR Analysis

Hepatic stellate cell line T6 cells with a density of 2 × 10^5^ cells/well were evenly spread and incubated in a six-well plate for 24 h and treated with 10% (v/v) concentrations of XCHT compound serum for a further 60 h. The total RNA of HSCT6 Cell was also isolated by using Trizol (TAKARA, Dalian, China). The nucleotide sequences of the primers were synthesized by TAKARA Biological Engineering Company. The nucleotide sequences of the primers used in this experiment were listed in Table 1. The results were analyzed by relative quantification of gene expression method (Xu et al., 2012).

**Table 1:** The primer sequence for RT-PCR.

Gene	Sequence (5′–3′)	
	
	Forward primer	Reverse primer
Nrf2	TTGGCAGAGACATTCC CATTTGTA	GAGCTATCGAGTGACTG AGCCTGA
Keap1	CATCGGCATCGCCA ACTTC	GCTGGCAGTGTGACA GGTTGA
Nqo1	TGGAAGCTGCAGAC CTGGTG	CCCTTGTCATACATG GTGGCATAC
HO-1	AGGTGCACATCCGT GCAGAG	CTTCCAGGGCCGTAT AGATATGGTA
GCLC	CTGCACATCTACCA CGCAGTCA	ATCGCCGCCATTC AGTAACAA
GCLM	AGACCGGGAACCTG CTCAAC	GATTTGGGAGCTCCA TTCATTCA
B-actin	GGAGATTACTGCCCTGG CTCCTA	GACTCATCGTACTCCTG CTTGCTG


### Western Blot Analysis

Hepatic stellate cell line T6 cells with a density of 2 × 10^5^ cells/well were evenly spread and incubated in a six-well plate for 24 h and treated with 10% (v/v) concentrations of XCHT compound serum for a further 60 h. Cytoplasmic and nuclear protein of each group were extacted by NE-PER Nuclear and Cytoplasmic Extraction Reagents (Thermo-Scientific, Rockford, United States). The cells were homogenized in RIPA buffer. Equal amounts of protein (15 μg) were separated on 8% SDS-polyacrylamide gel. The blots were incubated over-night with each protein anti-body (1:1000), washed, incubated with anti-rabbit IgG (1:1.000), anti-mouse IgG (1:1.000), and developed using ECL Western Blotting Substrate. Western blot signals were quantified using imager (Fusion-Fx-7 with BD-Software, Peqlab, Erlangen, Germany).

### Preparation of siRNA, Construction of siRNA Expression Vector and Transfection Assay

Nuclear factor erythroid 2-related factor 2-siRNA was designed and synthesized by Guangzhou *RiboBio* corporation. The Nrf2-siRNA sequences are shown in Table 2.

**Table 2:** Nrf2-siRNA sequences.

Plasmid constructs	Target sequence in mRNA(5′–3′)
si-r-Nfe2l2- 001(siRNA001)	CGAGAAGTGTTTGACTTTA
si-r-Nfe2l2- 002(siRNA002)	GGCAGAGACATTCCCATTT
si-r-Nfe2l2- 003(siRNA003)	GGATGAAGAGACCGGAGAA


*Nfe2l2: nuclear factor, erythroid 2-like 2/also called Nrf2, NF-E2-related factor 2.*

Cell viability assay, immunofluorescence assay, enzyme-linked immune assay, western blot analysis, and immune fluorescence assay of transfected cells were measured by the same method above.

### Statistical Analysis

All Data were expressed as mean ± standard deviation. The significance of differences between groups was evaluated by one-way ANOVA and *P* < 0.05 was considered as statistically significant differences.

## Results

### Effect of Different Concentration of XCHT Compound Serum on Proliferation of HSCT6 Cells

**FIGURE 1:**
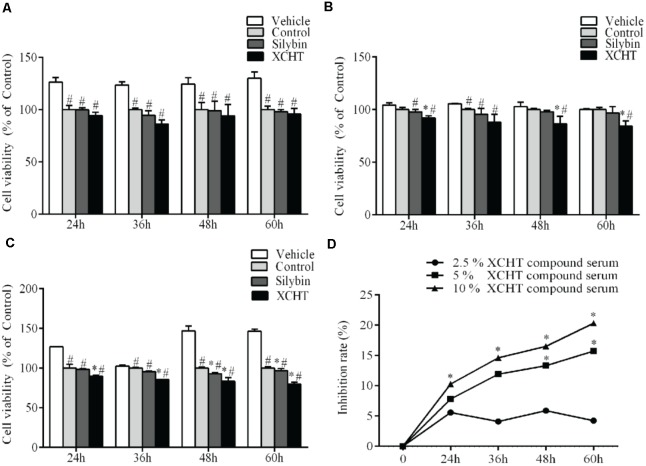
Effect of XCHT compound serum for 24, 36, 48, and 60 h on HSCT6 cells proliferation. **(A)** 2.5% compound serum **(B)** 5% compound serum **(C)** 10% compound serum **(D)** 2.5, 5, and 10% XCHT compound serum inhibition rate in 24, 36, 48, and 60 h. Data are shown as the mean ± SD (*n* = 4) of one representative experiment. ^#^*p* < 0.05 vs. the Vehicle control group, ^∗^*p* < 0.05 vs. the Control group.

In order to investigate the effect of XCHT compound serum in HSCT6 cells proliferation, cell viability was assessed with CCK8. Compared to the control group, 2.5% (v/v) concentration of XCHT compound serum treatment didn’t decrease cell viability (Figure 1A). However, 5 and 10% (v/v) concentration of XCHT compound serum treatment inhibited the proliferation of HSCT6 cells in 24, 48, and 60 h (Figures 1B,C). The highest inhibition rate of XCHT compound serum was 20.30% (Figure 1D). Therefore, 10% (v/v) concentration of XCHT compound serum were used for all subsequent experiments. These results suggested that XCHT compound serum could inhibit HSCT6 cells proliferation.

### Effect of XCHT Compound Serum on Collagen-I Secretion of HSCT6 Cells

**FIGURE 2:**
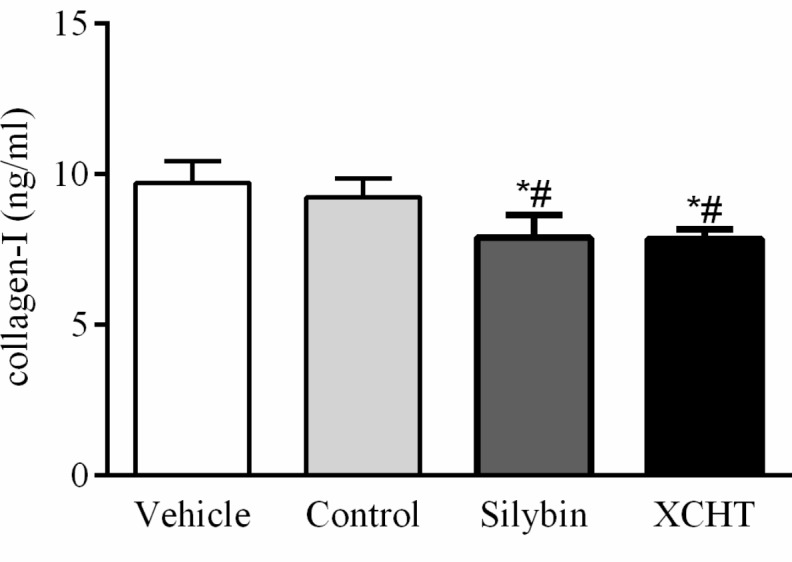
Effect of XCHT compound serum on collagen-I content in HSCT6 cells cultrue supernatant. Data are shown as the mean ± SD (*n* = 5) of one representative experiment. ^#^*p* < 0.05 vs. the Vehicle group, ^∗^*p* < 0.05 vs. the Control group.

In order to investigate the effect of XCHT compound serum on HSCT6 cells activation, concentration of collagen-I in the supernatant of each group were assessed with ELISA (Enzyme-linked immunosorbent, Jianglai bio, Shanghai, China) assay. The ELISA assay results indicated that 10% (v/v) concentration of XCHT compound serum treatment for 60 h decreased collagen-I secretion compared to control group (Figure 2).

### The Protein Expression of α-SMA in HSCT6 Cells

**FIGURE 3:**
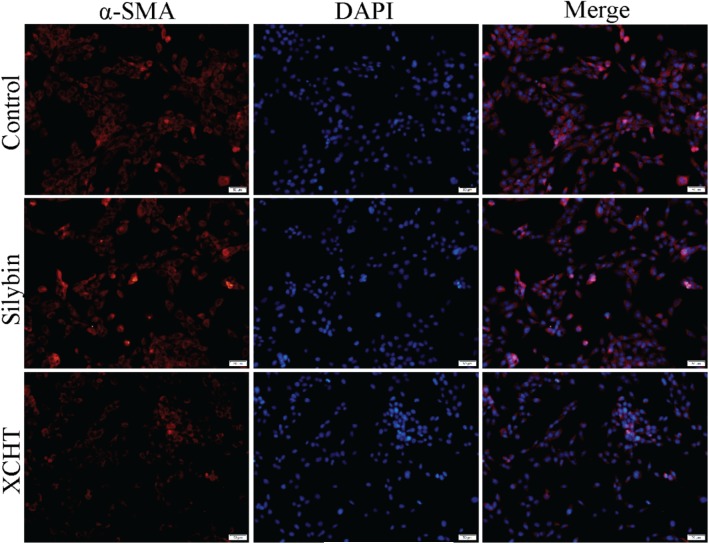
Effect of XCHT compound serum on the expression of α-SMA in HSCT6 cells.

Immunofluorescence (IF) experiment was used to evaluate the impact of XCHT compound serum on the expression of α-SMA. HSCT6 cells were fully stained with α-SMA in the control group (Figure 3). After treatment with XCHT compound serum for 24 h, the expression of α-SMA was reduced (Figure 3).

### Effect of XCHT Compound Serum on the Expression of Nrf2

**FIGURE 4:**
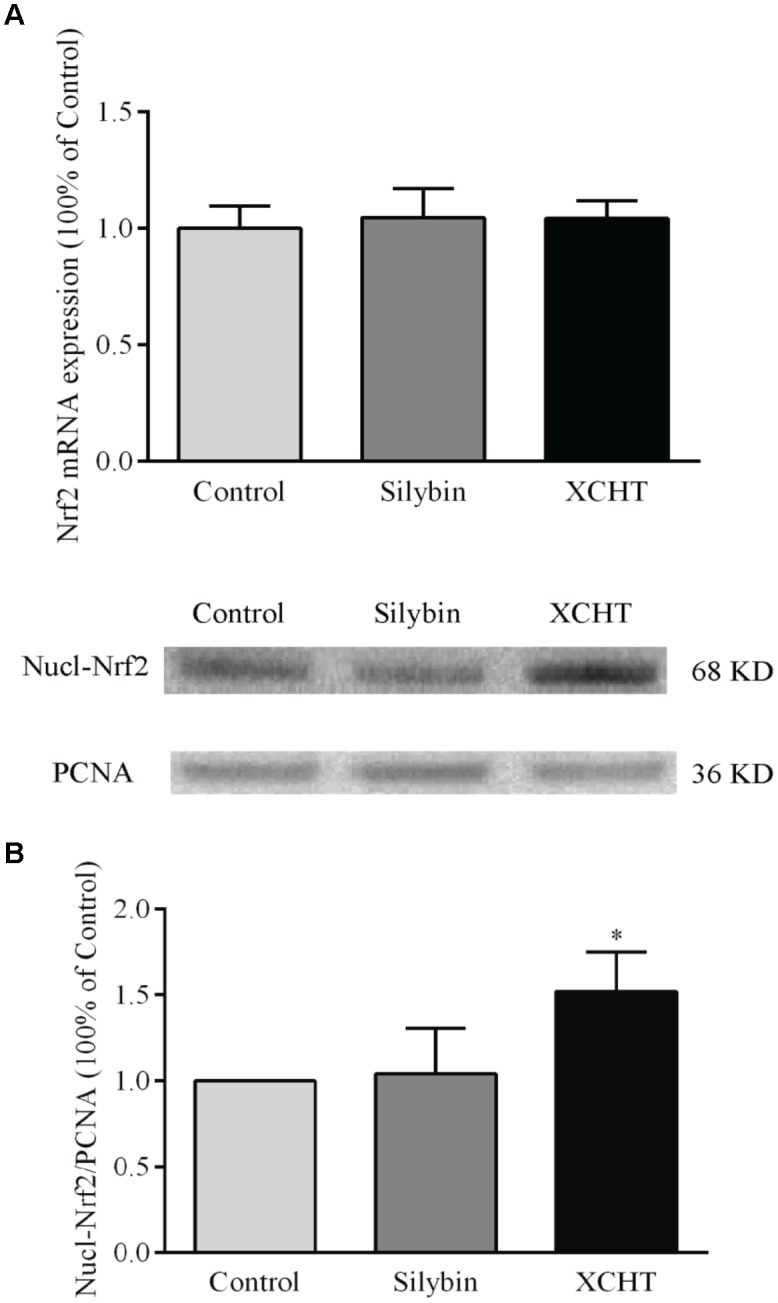
Effect of XCHT compound serum on the expressions of Nrf2 in HSCT6 cells. **(A)** The mRNA expression of Nrf2. **(B)** The protein expression of Nucl-Nrf2. Data are shown as the mean ± SD (*n* = 4) of one representative experiment. ^∗^*P* < 0.05 vs. the Control group.

**FIGURE 5:**
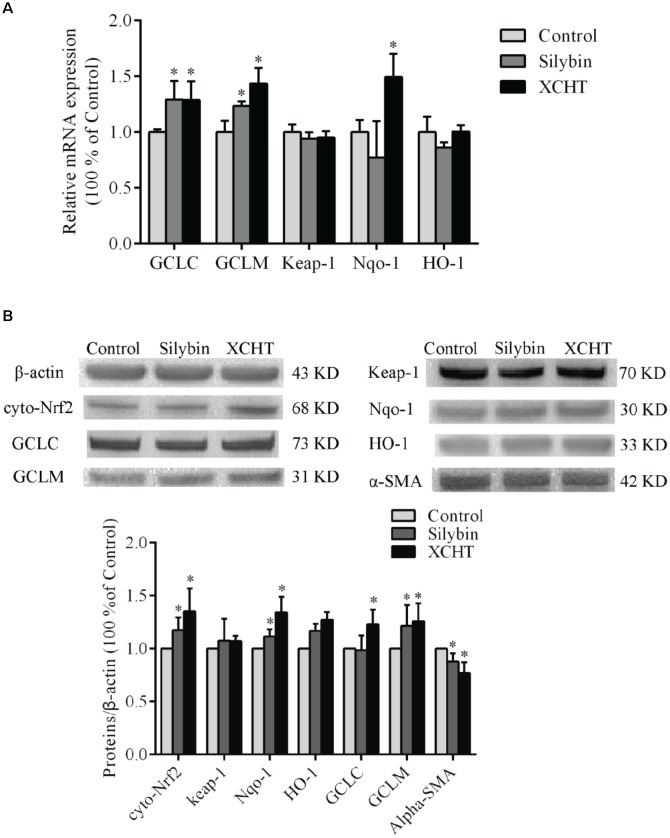
Effect of XCHT compound serum on the expressions of Keap-1, Nqo-1, HO-1, GCLC, and GCLM in HSCT6 cells. **(A)** The mRNA expression of Keap-1, Nqo-1, HO-1, GCLC and GCLM. **(B)** The protein expression of Cyto-Nrf2, Keap-1, Nqo-1, HO-1, GCLC and GCLM. Data are shown as the mean ± SD (*n* = 5) of one representative experiment. ^∗^*P* < 0.05 vs. the Control group.

There was no obvious change in Nrf2 gene expression after treated with XCHT compound serum or Silybin compound serum (Figure 4A). This illustrated that XCHT Compound serum couldn’t affect the transcription of Nrf2. But XCHT Compound serum could affects the translation and post-translational modification or protein localization of Nrf2 protein. The results of our Western Blot showed that XCHT increased Nrf2 protein levels in HSCT6 cell nucleus (Figure 4B) and Cytoplasm (Figure 5).

### Effect of XCHT Compound Serum on the Expression of α-SMA, GCLC, GCLM, HO-1, Keap-1 and Nqo-1

The mRNA expression of GCLC, GCLM, and Nqo-1 was significantly increased after treatment with XCHT compound serum for 60 h (Figure 5A). Similarly, the mRNA expression of GCLC and GCLM was significantly increased after treatment with Silybin compound serum for 60 h (Figure 5A). However, there was no distinct difference observed on the mRNA expression of Keap-1 and HO-1 mRNA among these groups.

After treatment with XCHT compound serum or Silybin compound serum for 60 h, α-SMA protein expression was significantly decreased in HSCT6 cells (Figure 5B) XCHT compound serum increased Nrf2 protein level in HSCT6 cell cytoplasm. The protein level of GCLC, GCLM, and Nqo-1 was significantly increased in XCHT group (Figure 5B). However, there was no difference between the groups in the expression of Keap-1 and HO-1 protein. These results suggested that XCHT compound serum could activate Nrf2 pathway.

### Transfection With Nrf2-siRNA

**FIGURE 6:**
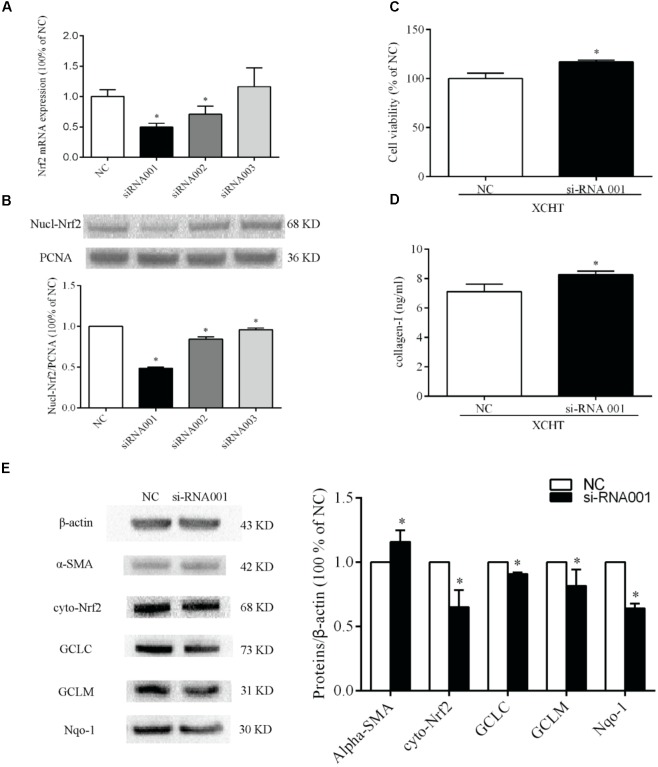
Nrf2-siRNA transfection. Selection of Nrf2 mRNA sequence target **(A)** Semiquantitative analysis of the RT-PCR result **(B)** Semiquantitative analysis of the western blotting results. **(C)** The proliferation was increased in tansfected cells after treat with XCHT compound serum. **(D)** The content of collagen-I was increased in tanfected cells after treat with XCHT compound serum. **(E)** Effect of XCHT compound serum on protein level of α-SMA, Nrf2, Nqo-1, GCLC, and GCLM in tansfected cells. ^∗^*p* < 0.05 vs. NC group. Data are shown as the mean ± SD (*n* = 3) of one representative experiment.

There were three Nrf2-siRNA sequences (Table 2). These sequences were transfected into HSCT6 cells using *riboFECTTMCP*. After transfected for 24 h, the level of Nrf2 mRNA in siRNA001 group was reduced to 49.98% of NC group (Figure 6A). After transfected for 60 h, the level of Nrf2 protein in siRNA001 group was reduced to 48.65% of NC group (Figure 6B). Therefore, we chose siRNA001 for the subsequent experiments.

### Effect of XCHT Compound Serum on Protein Level of α-SMA, Nrf2, Nqo-1, GCLC, and GCLM in Transfected HSCT6 Cells

The results showed that protein levels of Nrf2, Nqo-1, GCLC, and GCLM were significantly decreased when compared to NC group (Figure 6E). This result indicates that siRNA001 downregulated Nrf2 protein level. Compared to NC group, the protein level of α-SMA was significantly increased after treated with XCHT compound serum (Figure 6E).

### Effect of XCHT Compound Serum in the Proliferation of Transfected HSCT6 Cells

Compared to the NC group, XCHT compound serum treatment for 60 h increased cell viability in transfected cells (Figure 6C).

### Effect of XCHT Compound Serum on the Collagen-I Secretion of Transfected HSCT6 Cells

The result showed that XCHT compound serum decressed the secretion of Collagen-I in HSCT6 cells (Figure 2). In transfected cells, however, XCHT compound serum increased the amount of Collagen-I compared with NC group (Figure 6D).

### Effect of Nrf2 siRNA on α-SMA Protein Expression in HSCT6 Cells

**FIGURE 7:**
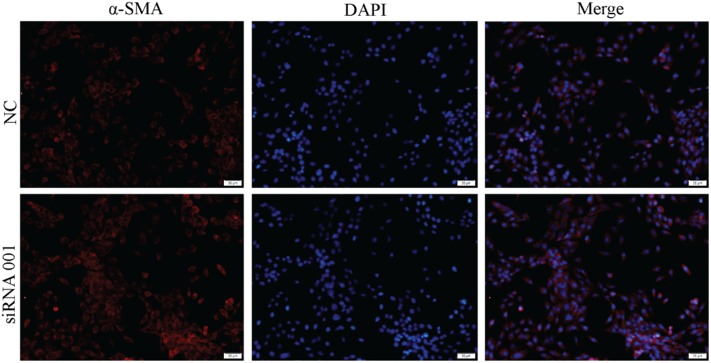
Effect of XCHT compound serum on the expression of α-SMA in transfected cell.

The result showed that XCHT compound serum reduced α-SMA fluorescent staining (Figure 3). In transfected cells, however, XCHT compound serum increased α-SMA fluorescent staining compared with NC group (Figure 7).

## Discussion

Previous studies in our laboratory had proved that XCHT could be used to treat hepatic fibrosis in rats (Li et al., 2014a,b,c, 2017; Hu et al., 2018). Considering HSCs proliferation and activation are the critical factor during liver fibrosis (Rockey, 2000; Fagone et al., 2015; Romanelli and Stasi, 2016), hepatic stellate cell line T6 cells were used in this study. Our results demonstrated that XCHT compound serum successfully inhibited HSCs proliferation and activation, as evidenced by (1) It was found that cell viability was significantly inhibited after XCHT or Silybin compound serum treatment compared with control group. (2) The amount of collagen-I was decreased after XCHT or Silybin compound serum treatment for 60 h compared with control group. (3) The production of α-SMA decreased after XCHT or Silybin compound serum treatment for 24 h compared with control group. While α-SMA is a symbol of the myofibroblasts. These results indicated that XCHT compound serum inhibited HSCs proliferation and activation.

HSCs were identified for the first time by von Kupffer in 1876. HSCs are localized in the subendothelial space of disse, interposed between liver sinusoidal endothelial cells (LSECs) and hepatocytes, and represented about 10% of all resident liver cells (Wake, 1971). HSCs maintain a non-proliferative and quiescent phenotype in normal liver. However, HSCs were activated when liver was injured or cultured *in vitro*. HSCs transdifferentiated from vitamin A-storing cells to myofibroblasts, which are characterized by enhancing ECM production (Puche et al., 2013). Inhibition of HSCs proliferation and activation can delay or even reverse liver fibrosis. Present study showed that 5 and 10% (v/v) concentration XCHT compound serum could significantly inhibit the proliferation of HSCT6 cells. Furthermore, XCHT compound serum significantly inhibited HSCs activation, as evidenced by the results of collagen-I and α-SMA. These findings illustrated the impact of XCHT compound serum on anti-HSCT6 cells activation.

Oxidative stress refers to redundant high reactive molecules (ROS, reactive oxygen species, and RON, reactive nitrogen free radicals) and decreased ability to remove, leading to redox system imbalance, and causing cell oxidative damage (Bartz and Piantadosi, 2010; Valavanidis et al., 2013). Nrf2 pathway is the key pathway of cellular antioxidant stress. Antioxidant enzymes regulated by this signal pathway can eliminate ROS and other harmful substances, thus showing the role of detoxification and neutralization (Kundu and Surh, 2010). Under normal conditions, Nrf2 binds to cytoplasmic protein Keap1 in the cytoplasm. Nrf2 and Keap1 are separated by external stimuli, and Nrf2 migrates to the nucleus and accumulates in the nucleus and binds with the ARE. Downstream target genes such as HO-1, Nqo1, GCLM, and GCLC were activated to resist oxidative stress. Our previous study (Li et al., 2017) demonstrated that oxidative stress was closely related to liver fibrosis. The results of this study showed that XCHT compound serum decreased the expression of α-SMA and the amount of Collagen-I in HSCT6 cells. These results were consistent with our previous work *in vivo*. Furthermore, the expressions of GCLC, GCLM, and Nqo-1 were increased by XCHT compound serum both on mRNA and protein levels. Although XCHT did not affect the expression of Nrf2 mRNA in HSCT6 cells. But XCHT compound serum affects the translation and post-translational modification or protein localization of Nrf2 protein. The results of our Western Blot showed that XCHT compound serum increased Nrf2 protein levels in HSCT6 cell nucleus and cytoplasm. And the elevated Nrf2 expression could promote GCLC, GCLM, and Nqo-1 mRNA level. There are some relevant studies that have proved it (Furfaro et al., 2016; Xue et al., 2017). We consider that XCHT compound serum affects the transcription and translation of other genes downstream of Nrf2 pathway by regulating Nrf2. Therefore, XCHT compound serum may inhibit HSCT6 cells proliferation and activation through its antioxidant effect.

In this study, the relationship between the antioxidant effect of Nrf2 pathway and the effect of XCHT against hepatic fibrosis were further clarified. siRNA has been widely utilized for study of gene function and gene therapy (Jackson et al., 2006; Tschuch et al., 2008). These Nrf2-siRNA sequences were designed by the *Ribobio* corporation. In this study, siRNA001 was identified the interference effect. More results showed that the effect of XCHT compound serum inhibiting HSCT6 cells proliferation was decreased after Nrf2 down-regulation in HSCT6 cells by siRNA001. Furthermore, the expression of a-SMA and the amount of Collagen-I were increased after XCHT compound serum treatment compared with NC group, suggesting the effect of XCHT compound serum inhibiting HSCT6 cells activation was decreased. Taking together, these results suggested that Nrf2 pathway played a significant role in XCHT compound serum in inhibiting HSCT6 cells proliferation and activation.

In summary, XCHT compound serum inhibited HSCT6 cells proliferation and activation. The results of the study indicated a significant functional role for Nrf2 in the development of liver fibrosis, and upregulating Nrf2 expression with XCHT compound serum could be an effective way to treat liver fibrosis.

## Ethics Statement

Rats were kept in a SPF-grade animal Facilities (Certificate No. SYXK 2011-004) at Zunyi Medical University. All animal care and experimental protocols were complied with the Animal Management Guidelines of the Chinese Ministry of Health.

## Author Contributions

JL, S-fX, and S-yY participated in research design. RH, W-yJ, and Z-wZ performed the experiments, analyzed the data, and wrote the manuscript. S-fX, ZX, and RH performed the data analysis. JL, S-yY, and ZX amended the manuscript.

## Conflict of Interest Statement

The authors declare that the research was conducted in the absence of any commercial or financial relationships that could be construed as a potential conflict of interest.

## Supplementary Material

Figure S1 Content of baicalin in the combination granular state of XCHT.
