# Supplementary figures and images for: Xiaochaihutang Inhibits the Activation of Hepatic Stellate Cell Line T6 Through the Nrf2 Pathway
Source: Front Pharmacol. 2019 Jan 7;9:1516. doi: 10.3389/fphar.2018.01516 (PMC6330344; doi:10.3389/fphar.2018.01516)

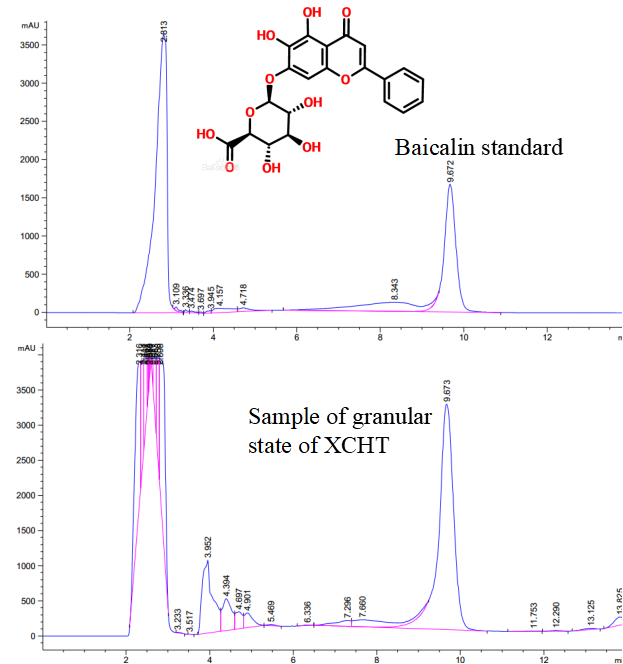

Supplement: Figure S1 — Content of baicalin in the combination granular state of XCHT. [file Image_1.TIF]
